# Structural and Color Alterations of Teeth following Orthodontic Debonding: A Systematic Review

**DOI:** 10.3390/jfb15050123

**Published:** 2024-05-10

**Authors:** Francesco Inchingolo, Angelo Michele Inchingolo, Lilla Riccaldo, Roberta Morolla, Roberta Sardano, Daniela Di Venere, Andrea Palermo, Alessio Danilo Inchingolo, Gianna Dipalma, Massimo Corsalini

**Affiliations:** 1 Department of Interdisciplinary Medicine, University of Bari “Aldo Moro”, 70121 Bari, Italy; a.inchingolo3@studenti.uniba.it (A.M.I.); l.riccaldo@studenti.uniba.it (L.R.); roberta.morolla@uniba.it (R.M.); roberta.sardano@uniba.it (R.S.); daniela.divenere@uniba.it (D.D.V.); ad.inchingolo@libero.it (A.D.I.); massimo.corsalini@uniba.it (M.C.); 2 College of Medicine and Dentistry, Birmingham B4 6BN, UK; andrea.palermo2004@libero.it

**Keywords:** orthodontic debonding, enamel appearance, brackets, retainer, crack, roughness, discoloration

## Abstract

Aim: The objective of this study was to explore the effects of fixed orthodontic appliances on enamel structure by assessing microfractures, surface roughness, and alterations in color. Methods: This review followed the Preferred Reporting Items for Systematic Reviews and Meta-Analyses guidelines. A systematic search of online databases was conducted using the keywords ‘enamel’ AND ‘orthodontic debonding’. Eligibility criteria included both in vivo and ex vivo clinical trials conducted on human teeth. Results and Discussion: A total of 14 relevant papers were analyzed. Various instruments and techniques were utilized across different studies to assess surface roughness, color change, and surface fractures. Conclusions: The findings of this study suggest that ceramic brackets may lead to an increase in enamel fractures, particularly during bracket removal. The surface roughness of enamel exhibits variability depending on the adhesive substance and polishing methods used post-removal. Fixed orthodontic appliances could induce changes in enamel color, which may be alleviated by the use of nano-hydroxyapatite or specific polishing techniques. Further research is necessary to identify effective strategies for managing these color changes and improving the overall outcomes of fixed orthodontic treatment.

## 1. Introduction

The field of clinical orthodontic procedures has experienced notable evolution with the introduction of bracket bonding onto the enamel surface. This adhesive method not only accelerates the process considerably but also facilitates more accurate positioning of attachments on specific teeth, thus improving patient comfort [1].

The adhesive technique utilizes the bonding capability of the adhesive to establish a connection between the enamel surface and the base of the attachment [2].

While the aim is for this type of bonding to remain robust throughout orthodontic treatment, it often does not. Masticatory forces or inadequate isolation of the tooth during bonding are two common reasons for brackets to detach from teeth [3,4,5,6].

The removal of orthodontic attachments is a primary concern for clinicians during orthodontic treatment. The objective of this final step is to fully eliminate the composite and adhesive from the enamel surface, thereby restoring the enamel to its original condition [7,8,9,10,11,12].

The separation forces exerted by orthodontic pliers can lead to the development of fracture lines and/or enamel fissures, particularly when ceramic brackets debond [13,14,15,16,17,18].

To prevent irreversible iatrogenic harm like enamel surface loss, enamel cracks, pulp necrosis, or lingering adhesive remnants, it is vital to employ an appropriate debonding approach [19,20,21,22].

The gentleness of the debonding process is affected by various factors, including:

- The type of brackets used.
- The instruments used for bracket removal and the technique applied.
- The tools used for removing residual adhesive remnants from the enamel surface.

### 1.1. Type of Brackets Used

Frequently, the orthodontist’s choice to use ceramic brackets over metal ones is driven by the patient’s preference for aesthetics [23,24]. Ceramics possess inherent characteristics that lead to clinical limitations, including high brittleness (low resistance to fracture) and challenges in forming chemical bonds with adhesive materials [25,26,27,28].

Odegaard illustrated how ceramic attachments often detach at the enamel–adhesive substance contact [29,30]. Separation from metal attachments, conversely, usually happens at the interface between the bracket and adhesive substance, posing a lower risk to the integrity of the enamel [31,32,33].

### 1.2. The Tools Used for Attachment Removal

In contemporary practice, a variety of debonding techniques are utilized. Mechanical, thermal, and ultrasonic methods represent some examples. Mechanical debonding involves employing different types of forceps like How’s forceps, Weingart’s forceps, straight cutters, or specialized debonding forceps. Research has shown that the latter is the most effective in minimizing damage to the enamel surface.

Thermal debonding presents an alternative for removing ceramic attachments by utilizing the heat generated by a laser, reducing the force required to detach the brackets. However, drawbacks of this method include the bulky nature of the handpiece, the potential irritation to surrounding mucosal tissues, and the discomfort caused by increased warmth [34,35].

Ultimately, studies have shown that ultrasonic debonding drastically decreases adhesion strength, reducing it from 9.2 MPa to 0.28 MPa. However, drawbacks of this method include prolonged operation, leading to patient discomfort and the risk of causing pain if ultrasonic tips make contact with the ceramic attachment [36].

Despite the validity of all three debonding techniques, the mechanical one achieves much more satisfactory results.

### 1.3. The Technique Employed

**Figure 1:**
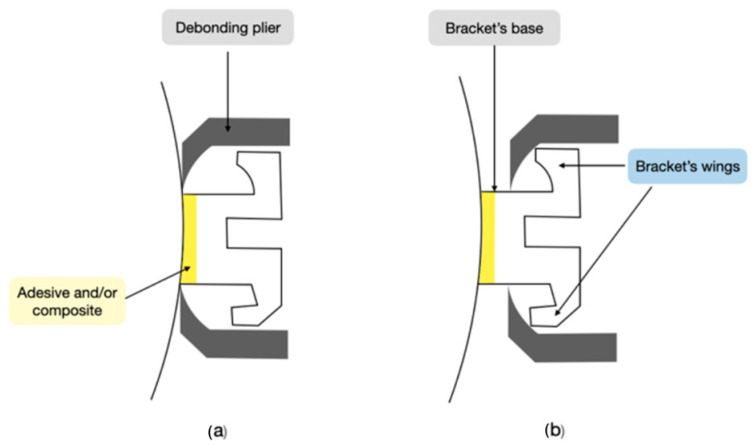
Two debonding techniques: (**a**) base method; (**b**) wings method. Figure created by author R.M.

The literature describes two debonding techniques: the wing approach and the base method (Figure 1). The wing approach involves gripping the mesial and distal wings of the bracket using forceps, whereas the base method requires inserting the forceps between the bracket’s base and the tooth surface. Brosh’s latest research indicates an overlap between these two debonding procedures [37].

### 1.4. The Instruments Used

Another relevant factor is the protocol for removing the adhesive material at the enamel level [38,39,40]. Even in present times, diverse viewpoints exist in the literature, but it is possible to draw the following conclusions:

- Debonding forceps or scalers are inadequate as they can result in significant enamel loss.
- Diamond burs cause permanent damage to enamel.
- Arkansas stones do not completely eliminate composite residue.
- Green stones alone are insufficient in fully removing bonding material.
- Ultrasonic methods necessitate extended chair time and may lead to the loss of small enamel fragments.
- It is recommended that a tungsten carbide bur is used for the primary removal of composite, followed by a disk to eliminate the remaining adhesive residue.

Theoretically, scratches and grooves on enamel could potentially lead to the development of stains and reduce resistance to organic acids found in plaque, thereby increasing the risk of demineralization for teeth [9,41,42].

Regardless, the debonding procedure almost always leads to some degree of enamel damage, even if it is not readily visible to the naked eye and might not always be clinically significant [43,44,45,46].

The objective of this systematic investigation was to analyze alterations in enamel color and roughness resulting from debonding, irrespective of the bracket type or removal techniques utilized. The aim was to explore the most efficient methods to prevent damage and improve the final appearance of enamel after orthodontic treatment. Due to limited research availability in the literature, clinical studies conducted both in vivo and on extracted teeth were assessed.

## 2. Materials and Methods

### 2.1. Protocol and Registration

This research adhered to the Preferred Reporting Items for Systematic Reviews and Meta-Analyses (PRISMA) guidelines and was registered with PROSPERO (International Prospective Register of Reviews) under registration number 473047 [47].

### 2.2. Search Processing

On 15 September 2023, searches were conducted across the PubMed, Scopus, and Web of Science databases using the Boolean keywords “enamel” AND “orthodontic debonding” as part of our search strategy, for an analysis of texts from the past 10 years (2013–2023). These keywords were selected as they closely aligned with the objective of our study, which aimed to assess changes in enamel color and roughness resulting from debonding, irrespective of the bracket type or removal procedures employed. The primary focus was to explore the most effective approaches for minimizing damage and enhancing the overall appearance of enamel following orthodontic treatment.

### 2.3. Eligibility Criteria and Study Selection

The evaluation of the title and abstract, as well as the entire content, were the two phases of the selection procedure. Any item that met the following parameters was considered: (a) in vivo and ex vivo clinical studies; (b) free full text; (c) human participants of any age; and (d) English language. Publications that lacked original data (such as meta-analyses, research techniques, conference presentations, in vitro or animal studies) were excluded. The preliminary search yielded titles and abstracts that were appraised for relevance. Full publications from relevant studies were obtained for additional examination. Two distinct reviewers (R.M. and L.R.) evaluated the retrieved studies for inclusion using the aforementioned criteria.

### 2.4. Data Processing

Two reviewers, R.M. and L.R., independently assessed the quality of the studies based on selection criteria that fit the purpose of the review, after conducting a database search to extrapolate the findings. The selected articles were downloaded in the 6.0.15 version for usage with Zotero. To resolve any differences between the two writers, a senior reviewer (F.I.) was contacted. The screening process allowed the exclusion of any publications that did not fit the themes examined. After being found to meet the predefined inclusion criteria, the full text of the publications was read.

### 2.5. PICOS Requirements

The PICOS (Population, Intervention, Comparison, Outcome, Study Design) criteria, which were used in this evaluation, encompass population, intervention, comparison, outcomes, and study design (Table 1).

#### Quality Assessment

The quality of the included papers was assessed by two reviewers, R.F. and E.I., using the ROBINS, which is a tool developed to assess risk of bias in the results of non-randomized studies that compare health effects of two or more interventions. Seven points were evaluated and each was assigned a degree of bias. A third reviewer (FI) was consulted in the event of a disagreement until an agreement was reached.

## 3. Results

### 3.1. Selection and Characteristics of This Study

**Figure 2:**
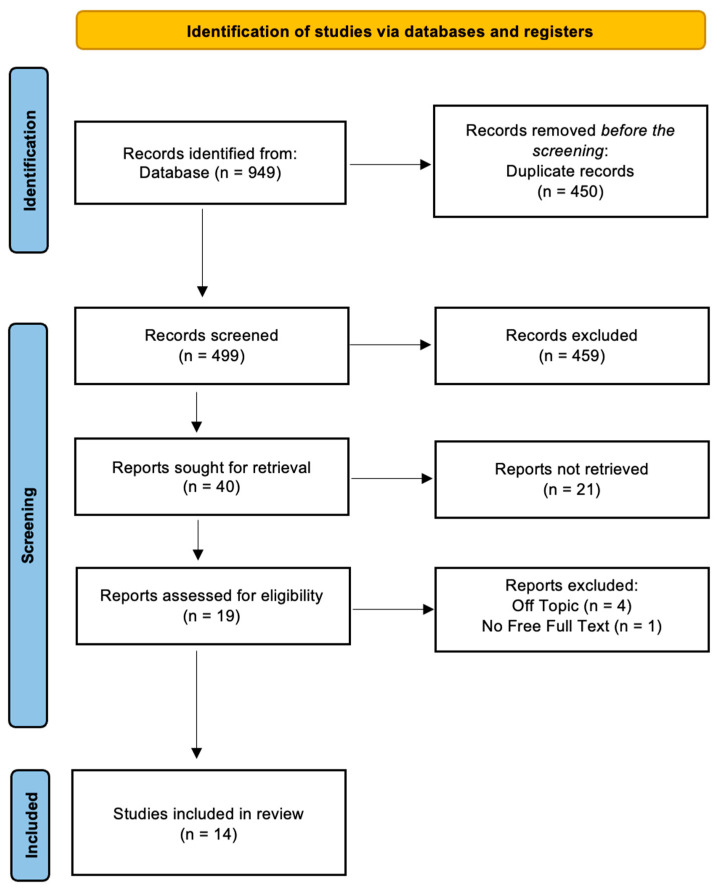
PRISMA ScR flowchart diagram of the inclusion process.

A total of 949 papers were found in the online databases (Web of Science *n* = 282, PubMed *n* = 337, and Scopus *n* = 329) using the Boolean keywords “enamel” AND “orthodontic debonding” as the search string; After removing 450 duplicates, 499 studies were taken into consideration by reading the title and abstract, focusing on the different techniques and the variation in enamel structure after the debonding procedure. This analysis led to the selection of 40 records out of 499 papers, whereas 459 articles were excluded for different reasons (229 off topic, 20 reviews, 150 in vitro, 20 on animals). Subsequently, 21 non-retrieved records were excluded because texts were not available. After reading the full texts of the remaining 19 reports eligible, 5 more reports were excluded (1 was not free and 4 were off topic). Finally, 14 papers were chosen for the systematic review. Figure 2 summarizes the selection procedure. The study data were selected by analyzing the study design, patients, type of intervention, and outcomes (Table 2).

#### Quality Assessment and Risk of Bias

**Figure 3:**
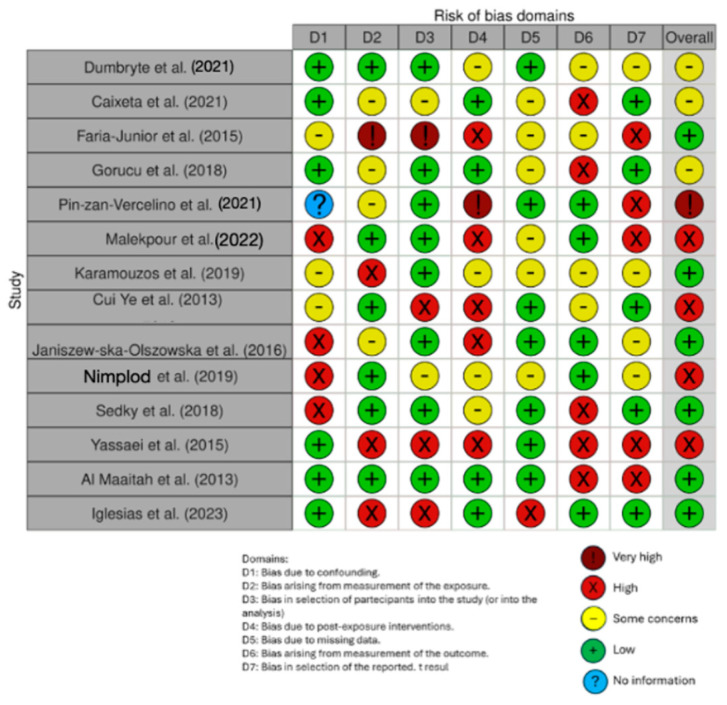
Evaluation of bias by ROBINS: Dumbryte et al. (2021) [48]; Caixeta et al. (2021) [49]; Faria-Junior et al. (2015) [50]; Gorucu et al. (2018) [51]; Pin-zan-Vercelino et al. (2021) [52]; Malekpour et al. (2022) [53]; Karamouzos et al (2019) [54]; Cui Ye et al. (2013) [55]; Janiszew-ska-Olszowska et al. (2016) [56]; Nimplod et al (2019) [57]; Sedky et al (2018) [58]; Yassaei et al. (2015) [59]; Al Maaitah et al. (2013) [60]; Iglesias et al. (2023) [61].

The risk of bias in the included studies is reported in Figure 3. Regarding the bias due to confounding, most studies had a high risk. The bias arising from measurement was a parameter with a low risk of bias. Many studies had a low risk of bias due to bias in their selection of participants. Bias due to post-exposure could not be calculated due to high heterogeneity. The bias due to missing data was low in many studies. Bias arising from measurement of the outcome was low. Bias in the selection of the reported results was high in most studies. The final results showed that 10 studies had a high risk of bias, 3 had a very high risk of bias, and 8 had a low risk of bias.

## 4. Discussion

The application of fixed orthodontic devices requires a tooth surface preparation procedure that inevitably modifies the enamel structure. This alteration includes the formation of cracks, the loss of the fluoride-rich surface layer, the presence of residual adhesive residue, and surface roughening, leading to the accumulation of plaque [62,63,64,65,66].

### 4.1. Microcracks

Due to the challenging clinical detection of enamel fractures, controlled surface cracks were intentionally created before attaching brackets to explore the impact of debonding shear stress on cracks associated with both metal and ceramic brackets, with or without indentations. Despite utilizing the same loading method, variations were observed in the size and configuration of the controlled microcracks in this investigation, encompassing features such as crack branching, fracture bridging, and crack bifurcation. This diversity might have been influenced by the distinct mechanical behaviors at the dentin–enamel junction-to-enamel surface and enamel prism orientations.

According to the studies, enamel fractures occurred in 13.3% of debonded ceramic brackets, particularly in samples exhibiting an exceptionally high debonding strength exceeding 40 MPa. The elevated shear bond strength noted in this investigation could be attributed to the preparation of the enamel surface. It seems that polishing enamel before installing or repositioning ceramic brackets should be avoided, as it led to a higher incidence of enamel fractures upon the removal of ceramic brackets compared to metal brackets.

The spontaneous repair of enamel microcracks is a common phenomenon in live teeth, safeguarding against crack propagation to the dentin and dental pulp, and this reparative process was also observed in extracted teeth, as mentioned in the studies [57,58,59,60,61,62,63,64,65,66,67,68].

In a clinical trial conducted in 2021, Dumbryte et al. investigated the presence of enamel microcracks (EMCs) on teeth during debonding. The study involved 13 participants who displayed visible EMCs before the initiation of therapy and 13 individuals who did not have EMCs before undergoing treatment with metal brackets. Following the debonding process, 25% of the teeth exhibited apparent EMCs. Notably, patients who had pre-existing EMCs before treatment experienced three times the level of cold sensitivity compared to those without EMCs prior to therapy [48,69,70].

### 4.2. Surface Roughness

Physiologically, the microstructure of teeth determines their micro-roughness [8,71].

In 2021, Caixeta et al. conducted an in vivo study involving 15 patients undergoing fixed orthodontic treatment. Brackets were bonded using traditional composite resin in some subjects and resin-modified glass ionomer cement (RMGIC) in others. Examination of epoxy resin models of the teeth before and after debonding revealed that, following polishing, both the composite and RMGIC groups exhibited comparable surface roughness (SR). The choice between composite and RMGIC materials did not impact enamel SR in either group, but the values were significantly reduced after polishing compared to the initial condition. In both instances, the smoother surfaces post-polishing were more apparent than at the beginning of the treatment [49,72].

In a study conducted in 2015, Faria-Junior et al. assessed the surface roughness (SR) and morphology of enamel after the polishing and removal of metal brackets, utilizing an SR tester and scanning electron microscopy. Ten orthodontic patients were included in the study. Upon completing their orthodontic treatment, metal brackets were removed, and a randomly selected tooth underwent finishing and polishing with either aluminum oxide disks or multi-laminated carbide burs. Dental replicas of teeth, both before and after polishing, were created using epoxy resin. Statistical methods were employed to analyze the data obtained from various SR measurements, and scanning electron microscopy was utilized to examine three samples from each group.

In comparison with aluminum oxide, the carbide bur group exhibited a significantly higher average roughness [50,73].

Using a confocal laser microscope at a magnification of 1080×, the buccal surfaces of 45 extracted human third molars were scrutinized, and 3D roughness characteristics were ascertained. Molar tubes were affixed after a 20 s etching, and the teeth were subsequently immersed in a 0.9% saline solution for 24 h before undergoing debonding. In 15 different specimens, any remaining adhesive was eliminated using a 12-fluted tungsten carbide bur, a one-step finisher and polisher, and adhesive residue remover. Subsequently, the analysis of surface roughness (SR) was repeated.

The roughness of enamel was found to increase due to orthodontic debonding and the removal of adhesive residue. The adhesive residue remover resulted in the smoothest surfaces, while the tungsten carbide bur yielded the roughest surfaces [56].

The assessment of the Adhesive Remnant Index (ARI) under the surgical microscope in Sedky et al.’s study revealed that employing the Er,Cr:YSGG laser (2.78 μm, 6 W, 20 Hz, 60 μs pulse duration) compared to traditional bracket removal techniques (using a specialized plier), despite necessitating longer application times and treatment durations, resulted in more effective bracket removal. This was achieved by minimizing the amount of adhesive residue and expediting the finishing and polishing phase [58].

In Yassaei’s study, the Er:YAG laser operating at 2.94 μm, 20 Hz, 125 mJ, and 2.5 W, and positioned at a distance of 5 mm, was utilized post-debonding with pliers to eliminate excess adhesive. This was compared to the use of a tungsten carbide bur or a zircon-rich glass-fiber-reinforced composite bur. Ninety teeth were treated using one of these three methods. SEM observations revealed that, contrary to findings reported by Almeida et al., the laser produced the most uneven surface after adhesive removal, while the composite bur resulted in the absolute smoothest surface [14,73,74,75,76]. While the tungsten carbide bur removed adhesive residue more rapidly than the other two methods, though not significantly faster, it caused a temperature increase of 5.5 °C at a significantly slower rate compared to the reinforced composite bur. The laser method resulted in the smallest temperature increase [59,77].

Forty-five extracted premolars were categorized into three groups based on the polishing bur used after debonding, with four specimens serving as the control group. A gold chain measuring 0.038 by 0.015 inches was bonded and subsequently extracted between the premolars. Group 1 utilized a white stone at high speed, Group 2 employed a high-speed handpiece with a 30-blade tungsten carbide bur, and Group 3 utilized a low-speed handpiece and a 30-blade tungsten bur to eliminate adhesive residue. All samples underwent examination using a confocal microscope to assess surface roughness (SR) characteristics after debonding and polishing.

The debonded enamel surface exhibited increased roughness compared to the enamel of control teeth, irrespective of the polishing bur used. There was variability in enamel roughness after debonding based on the polishing technique, with the 30-blade tungsten carbide bur, particularly when used at a low speed, resulting in a smoother surface. For the purpose of maintaining long-term teeth alignment, the binding strength between the tooth and the composite pad, as well as the retention wire, was deemed optimal. Given that each approach examined increased the SR of the treated enamel, further research into remineralization processes is warranted [61].

### 4.3. Color Changes

Enamel color alterations represent one of the most common and undesirable outcomes of fixed orthodontic treatment. The fluctuation in tooth surface color is intricately linked to the employed surface cleaning and polishing procedure designed to eliminate residual materials. Nevertheless, the use of various rotary instruments, particularly diamond burs in grinding, inevitably leads to enamel surface reduction and subsequent color changes [33,78,79].

In a clinical study conducted by Gorucu et al. in 2018, the appearance of enamel post-debonding was evaluated by categorizing a sample of 59 patients into four groups. Various enamel etching techniques and two distinct tungsten carbide burs were employed across these groups. The study’s results are noteworthy: despite the use of different etching and adhesive removal methods, observable and clinically undesirable changes in tooth color were noted following orthodontic treatment. Neither conventional nor self-etching techniques exhibited statistically significant differences in altering tooth color [51].

The introduction of increasingly high-performance polishing systems appears to yield promising outcomes. In their 2020 clinical study, Pinzan-Vercelino et al. assessed the surface roughness (SR) and color change of enamel following bracket detachment and polishing with two different systems. A 12-blade tungsten carbide bur on a low-speed handpiece was utilized for adhesive removal, followed by the application of Sof-Lex spiral wheels and disks in a random polishing process. Dental replicas were created using epoxy resin, polished, and attached with adhesive. Neither system significantly harmed dental enamel, as both color and roughness were equally affected. Both methods successfully restored the enamel, bringing it as close as possible to its initial state [52].

The 2022 study conducted by Malekpour et al. investigated the influence of nano-hydroxyapatite serum and various polishing and finishing methods on enamel discoloration resulting from post-orthodontic debonding procedures. The study compared the use of carbide burs alone (the standard procedure) with the combined use of carbide burs and Sof-Lex disks, followed by 10 days of nano-hydroxyapatite application post-debonding. Enamel coloration was then measured using a spectrophotometer. Clinically notable color changes were observed on average across all groups and intervals. Unlike Sof-Lex disks, which were found to significantly reduce tooth color changes shortly after debonding, the use of nano-hydroxyapatite showed no appreciable impact in terms of slowing these changes [53].

A total of 120 extracted teeth were subjected to coffee immersion after bracket removal. Subsequently, they underwent random polishing using four different techniques and were examined using a spectrophotometer. Teeth treated solely with a diamond bur exhibited a higher level of discoloration, attributed to increased aggressiveness and residual surface roughness (SR), leading to enhanced retention of potentially pigmented substances. The combination of the bur with other tools significantly reduced the discoloration rate. The most favorable outcomes were achieved when employing diamond burs in conjunction with polymerized urethane dimethacrylate resin, fine diamond powder, and silicon oxide (PoGo) cups, disks, and tips, followed by disks reverted by aluminum oxide. In contrast, the combination of burs and the OneGloss Polisher did not yield excellent results compared to the other two combined protocols [33,55].

Al Maaitah’s study aimed to evaluate the impact of fixed orthodontic treatment, etching techniques (self-etching and conventional), and various parameters (age, gender, tooth type) on tooth color. The Commission Internationale de l’Eclairage color scheme was employed to determine instrumental tooth shade before and after orthodontic treatment. The researchers identified statistically significant changes in color characteristics post-treatment, with teeth darkening and trending towards reddish–yellowish hues. The choice of etching technique (self-etching or conventional) did not affect the observed color alterations. Male patients and teenagers exhibited more pronounced color changes, possibly linked to their dietary habits, dental hygiene practices, and enamel characteristics [60,79].

The 2019 clinical study by Karamouzos et al. also showed that teeth undergo color change at the end of fixed orthodontic therapy; in particular, the greatest change was found during the first 3 months in teeth on which high-speed rotary instruments were used during debonding [48,54].

The studies that we reviewed had various limitations:

Heterogeneity in study designs: The studies analyzed encompass a diverse range of designs, including case–control, observational, retrospective, and prospective studies. Dental element analysis was conducted in vivo or after extraction. The incorporation of different designs may introduce heterogeneity, potentially compromising the reliability of the review’s findings.

Small sample sizes: Some of the included research has small sample sizes, which may limit the statistical power and generalizability of the results.

Lack of quality assessment: The review does not specify whether the included studies underwent quality assessment. Evaluating the methodological quality of the studies is crucial to determine the overall dependability of the evidence.

Limited scope of analysis: The review primarily focuses on enamel modification after debonding, analyzing various techniques and procedures. However, it does not address other potential complications affecting the tooth, such as variations in temperature within the pulpar chamber.

Further studies are essential to establish standardized and increasingly effective debonding protocols.

## 5. Conclusions

In conclusion, the use of fixed orthodontic appliances has several adverse effects on dental enamel, including the development of microcracks, surface roughness, and alterations in color. Ceramic brackets, particularly when excessive tooth surface preparation precedes bonding, are often linked to a higher incidence of enamel fracture.

Surface roughness in enamel can be affected by various factors, such as the type of adhesive material used and the polishing procedure after debonding. While the use of a low-speed tungsten carbide bur has shown effectiveness in reducing roughness, all polishing methods contribute to increased roughness, highlighting the need for further investigation into remineralization techniques.

Fixed orthodontic treatment may also result in undesirable changes in tooth color. Some studies have explored methods to mitigate these changes, such as the application of nano-hydroxyapatite or specific polishing systems, although success is not always guaranteed.

Age, gender, tooth type, and patient behaviors collectively influence the degree of color change during orthodontic treatment. These findings emphasize the importance of carefully evaluating the materials and techniques used in orthodontic procedures to minimize adverse effects on tooth enamel, such as microcracks, surface roughness, and color alterations. Further research is essential to discover approaches that mitigate these side effects while maintaining therapeutic effectiveness.

## Figures and Tables

**Table 1:** PICOS criteria.

Criterion	Application in the Present Study
Population	Young people and adults underwent fixed orthodontic treatment.
Intervention	Analysis of enamel changes after orthodontic debonding.
Comparisons	Comparison of different debonding techniques.
Outcomes	Best protocol to limit enamel alterations.
Study design	Clinical trials in vivo and ex vivo (extracted teeth).

**Table 2:** Descriptive summary of the included studies.

Authors	Study Design	Aim	Number of Patients/Teeth	Materials and Methods	Outcomes
Dumbryte et al. (2021) [48]	Clinical study	To determine whether teeth with and without visible enamel microcracks (EMCs) before the bonding procedure changed in the number of EMCs after having metal brackets removed.	26 patients	13 patients with teeth that had visible enamel microcracks (EMCs) before bonding and 13 subjects without teeth with EMCs were both included in the study. Before the application and after the removing of the bracket, the number of teeth with visible EMCs and the number of premolars without EMCs were counted for each subject twice, along with assessments of tooth sensitivity brought on by compressed air and cold testing.	Presence of EMCs in 25% of all teeth analyzed, regardless of whether these were already present before orthodontic treatment. Teeth with prior EMCs were also 3 times more sensitive to cold than others at the end of orthodontic treatment.
Caixeta et al. (2021) [49]	In vivo study	To measure the enamel surface roughness (SR) prior to and following the removal of brackets bonded to the maxillary central incisors with composite or RMGIC (resin-modified glass ionomer cement).	15 patients	Epoxy resin was used to create dental replicas both during the initial stages (prior to bonding) and after polishing with an aluminum oxide disk system. On the dental replicas, the Adhesive Remnant Index (ARI) and SR were measured, and data were analyzed.	The choice of composite or RMGIC material had no effect on how rough the enamel surface was, but polishing produced smoother surfaces than those that were initially present.
Faria-Junior et al. (2015) [50]	In vivo study	After removing metal brackets and polishing, an SR tester and scanning electron microscopy were used to assess the enamel’s morphology.	10 patients	After debonding, enamel SR was measured by making a comparison between teeth finished with aluminum oxide disks and those finished with multilaminated carbide burs.	The polishing system with an aluminum oxide disk produced less enamel roughness than the system with multilaminated carbide burs.
Gorucu et al. (2018) [51]	In vivo study	To compare the effects of different etching techniques, 12- and 24-bladed tungsten carbide burs, and polishing disks on tooth color changes during orthodontic treatment.	59 patients divided into 4 groups	Group 1: 37% phosphoric acid and adhesive primer; residual adhesives cleaned with 12-bladed tungsten carbide burs Group 2: 37% phosphoric acid and adhesive primer; residual adhesives cleaned with 24-bladed tungsten carbide burs Group 3: self-etch primer; residual adhesives cleaned with 12-bladed tungsten carbide burs Group 4: self-etch primer; residual adhesives cleaned with 24-bladed tungsten carbide burs	Regardless of the methods used for cleaning and preparing the enamel before orthodontic treatment, visible and clinically unacceptable tooth color changes still occurred.
Pinzan-Vercelino et al. (2021) [52]	Split-mouth randomized clinical trial	To compare the enamel SR and color alteration after bracket debonding and polishing using 2 systems.	36 patients	Comparison between Sof-lex disks and Sof-lex spiral wheels used for polishing after orthodontic debonding.	Both systems did not appear to significantly damage the enamel surface, and the color change was similar between them.
Malekpour et al. (2022) [53]	Randomized clinical trial	To assess the effect of nano-hydroxyapatite serum and different finishing and polishing techniques on color alterations of enamel caused by debonding procedures.	20 patients	Evaluation of color changes after the different techniques and follow-up.	The application of nano-hydroxyapatite had no significant effect in reducing tooth color changes after debonding.
Karamouzos et al. (2019) [54]	Split-mouth cohort study	To evaluate in vivo color modifications of teeth during retention, after removal of fixed orthodontic appliances.	48 patients	Evaluation of tooth color changes after debonding, and 3 months and 1 year later.	After fixed orthodontic treatment, teeth show long-term color change.
Cui Ye et al. (2013) [55]	Comparative study	This study compared the effects of four different enamel clean-up techniques to see if there were any differences in the degree of enamel discoloration after staining.	120 extracted premolars	120 teeth were randomly cleaned with one of four different techniques—carbide bur (TC), carbide bur and Sof-Lex polishers, carbide bur and Onegloss polishers, and carbide bur and PoGo polishers after being stored in coffee solution for seven days. The Crystaleye dental spectrophotometer was used to evaluate color both at the baseline and one week after being stored in a coffee solution.	The best results were obtained by combining the use of a diamond cutter with a polishing system. The three different systems showed no statistically significant differences.
Janiszewska-Olszowska et al. (2016) [56]	Observational study	To accurately quantify the degree of 3D enamel SR caused by the removal of any remaining orthodontic debonding adhesive from the molar tubes.	45 extracted third molars	Molar tubes were applied to the buccal surface of 45 extracted teeth, the teeth were placed for 24 h in a saline solution, and the tubes at a later time were removed. Subsequently, the surface was analyzed under a confocal laser microscope to assess its residual roughness.	The roughness of enamel is increased by orthodontic debonding and removal of adhesive residue. The adhesive residue remover produced the smoothest surfaces, and tungsten carbide bur produced the roughest.
Nimplod et al. (2019) [57]	Comparative study	This study assessed the degree of enamel fracture as well as the shear debonding strength of metal and ceramic brackets.	75 extracted premolars	75 human maxillary premolars were treated in different way: groups 1 and 2 were treated with bonding metal and ceramic brackets on polished enamel; groups 3 and 4 had brackets bonded on surfaces with created corner cracks; group 5 underwent an indentation procedure without bracket installation.	Even though ceramic brackets needed substantially more debonding force than metal brackets, debonding stress was only applied to the bonding site and did not affect the nearby enamel fissures.
Sedky et al. (2018) [58]	Comparative study	The purpose of this study was to compare the efficiency of the Er,Cr:YSGG laser with the traditional debonding approach in the removal of metal orthodontic brackets.	30 extracted premolars	The study involved a control group treated with conventional methods and a test group in which the brackets were removed using Er,Cr:YSGG lasering (2.78 μm, 6 W, 20 Hz, 60 μs pulse duration). SEM images were used to measure the ARI.	A lower ARI score was reported in the group treated with the laser.
Yassaei et al. (2015) [59]	Comparative study	This study compared the effects of three different residual adhesive removal techniques and assessed the best.	90 extracted premolars	Using bracket removal pliers, the brackets on 90 removed teeth were debonded. Through an access cavity, a thermocouple sensor was installed on the pulp chamber’s buccal wall to monitor heat changes while the adhesive was peeled off. Either a tungsten carbide bur, an erbium-doped yttrium aluminum garnet laser, or a fiber-reinforced composite bur was used to remove the adhesive residue from the enamel surface of the teeth. Images captured with a scanning electron microscope were used to evaluate how rough the enamel surface was. Additionally, the time taken to remove the adhesive was noted.	The smoothest enamel surface was produced using a fiber-reinforced composite bur, whereas the roughest was produced by an Er:YAG laser. Compared to the Er:YAG laser, tungsten carbide and composite burs produced greater heat. The devices that removed adhesive residue the fastest and slowest, respectively, were the tungsten carbide bur and Er:YAG laser.
Al Maaitah et al. (2013) [60]	Prospective clinical Ssudy	This study looked at how a self-etching primer and traditional acid etching affected the color of teeth after orthodontic treatment.	34 patients	Patients were divided into two age groups (adolescents and adults). Teeth color was measured with a spectrophotometer. The study calculated tooth color differences between pre-treatment and post-treatment, considering etching techniques, sexes, and age groups.	Teeth changed color as a result of fixed orthodontic appliances; the self-etching primer and traditional acid etching had comparable effects; men and adolescents experienced more color changes than women and adults.
Iglesias et al. (2023) [61]	Comparative study	This study examined several enamel polishing techniques.	45 patients	Forty-five healthy premolars underwent categorization into three groups based on the polishing bur utilized after debonding. Additionally, four specimens were designated as controls and underwent no intervention. Following the process of debonding and subsequent polishing, an examination of all samples was conducted using confocal microscopy to assess SR parameters.	A 30-blade tungsten carbide bur was used to polish the enamel, leaving a smooth surface.

## Data Availability

No new data were created or analyzed in this study. Data sharing is not applicable to this article.

## Abbreviations

ARI	Adhesive Remnant Index
EMC	enamel microcrack
RMGIC	Resin-modified glass ionomer cement
SR	surface roughness

## References

[1] Campbell P.M. (1995). Enamel Surfaces after Orthodontic Bracket Debonding. Angle Orthod..

[2] Herrera-Barraza V., Arroyo-Larrondo S., Fernández-Córdova M., Catricura-Cerna D., Garrido-Urrutia C., Ferrer-Valdivia N. (2022). Complications Post Simple Exodontia: A Systematic Review. Dent. Med. Probl..

[3] Thompson R.E., Way D.C. (1981). Enamel Loss Due to Prophylaxis and Multiple Bonding/Debonding of Orthodontic Attachments. Am. J. Orthod..

[4] Inchingolo F., Tatullo M., Abenavoli F.M., Marrelli M., Inchingolo A.D., Palladino A., Inchingolo A.M., Dipalma G. (2011). Oral Piercing and Oral Diseases: A Short Time Retrospective Study. Int. J. Med. Sci..

[5] Minervini G., Franco R., Marrapodi M.M., Di Blasio M., Ronsivalle V., Cicciù M. (2023). Children Oral Health and Parents Education Status: A Cross Sectional Study. BMC Oral Health.

[6] Osorio R., Toledano M., García-Godoy F. (1998). Enamel Surface Morphology after Bracket Debonding. ASDC J. Dent. Child..

[7] Eslamian L., Borzabadi-Farahani A., Tavakol P., Tavakol A., Amini N., Lynch E. (2015). Effect of Multiple Debonding Sequences on Shear Bond Strength of New Stainless Steel Brackets. J. Orthod. Sci..

[8] Fjeld M., Øgaard B. (2006). Scanning Electron Microscopic Evaluation of Enamel Surfaces Exposed to 3 Orthodontic Bonding Systems. Am. J. Orthod. Dentofac. Orthop..

[9] Patano A., Malcangi G., Sardano R., Mastrodonato A., Garofoli G., Mancini A., Inchingolo A.D., Di Venere D., Inchingolo F., Dipalma G. (2023). White Spots: Prevention in Orthodontics-Systematic Review of the Literature. Int. J. Environ. Res. Public Health.

[10] Minervini G., Franco R., Marrapodi M.M., Fiorillo L., Cervino G., Cicciù M. (2023). The Association between Parent Education Level, Oral Health, and Oral-Related Sleep Disturbance. An Observational Crosssectional Study. Eur. J. Paediatr. Dent..

[11] Ulusoy C. (2009). Comparison of Finishing and Polishing Systems for Residual Resin Removal after Debonding. J. Appl. Oral Sci. Rev..

[12] Zaher A.R., Abdalla E.M., Abdel Motie M.A., Rehman N.A., Kassem H., Athanasiou A.E. (2012). Enamel Colour Changes after Debonding Using Various Bonding Systems. J. Orthod..

[13] Eslamian L., Borzabadi-Farahani A., Mousavi N., Ghasemi A. (2012). A Comparative Study of Shear Bond Strength between Metal and Ceramic Brackets and Artificially Aged Composite Restorations Using Different Surface Treatments. Eur. J. Orthod..

[14] Ma T., Marangoni R.D., Flint W. (1997). In Vitro Comparison of Debonding Force and Intrapulpal Temperature Changes during Ceramic Orthodontic Bracket Removal Using a Carbon Dioxide Laser. Am. J. Orthod. Dentofac. Orthop..

[15] Janiszewska-Olszowska J., Tandecka K., Szatkiewicz T., Sporniak-Tutak K., Grocholewicz K. (2014). Three-Dimensional Quantitative Analysis of Adhesive Remnants and Enamel Loss Resulting from Debonding Orthodontic Molar Tubes. Head Face Med..

[16] Ryf S., Flury S., Palaniappan S., Lussi A., van Meerbeek B., Zimmerli B. (2012). Enamel Loss and Adhesive Remnants following Bracket Removal and Various Clean-Up Procedures In Vitro. Eur. J. Orthod..

[17] Patano A., Malcangi G., De Santis M., Morolla R., Settanni V., Piras F., Inchingolo A.D., Mancini A., Inchingolo F., Dipalma G. (2023). Conservative Treatment of Dental Non-Carious Cervical Lesions: A Scoping Review. Biomedicines.

[18] Giorgini E., Sabbatini S., Conti C., Rubini C., Rocchetti R., Fioroni M., Memè L., Orilisi G. (2017). Fourier Transform Infrared Imaging Analysis of Dental Pulp Inflammatory Diseases. Oral Dis..

[19] Almeida L.E., Cicciù M., Doetzer A., Beck M.L., Cervino G., Minervini G. (2023). Mandibular Condylar Hyperplasia and Its Correlation with Vascular Endothelial Growth Factor. J. Oral Rehabil..

[20] Minervini G., Franco R., Marrapodi M.M., Fiorillo L., Cervino G., Cicciù M. (2023). Post-traumatic Stress, Prevalence of Temporomandibular Disorders in War Veterans: Systematic Review with Meta-analysis. J. Oral Rehabil..

[21] Burapavong V., Marshall G.W., Apfel D.A., Perry H.T. (1978). Enamel Surface Characteristics on Removal of Bonded Orthodontic Brackets. Am. J. Orthod..

[22] Alessandri Bonetti G., Zanarini M., Incerti Parenti S., Lattuca M., Marchionni S., Gatto M.R. (2011). Evaluation of Enamel Surfaces after Bracket Debonding: An In-Vivo Study with Scanning Electron Microscopy. Am. J. Orthod. Dentofac. Orthop..

[23] Minervini G., Franco R., Marrapodi M.M., Almeida L.E., Ronsivalle V., Cicciù M. (2023). Prevalence of Temporomandibular Disorders (TMD) in Obesity Patients: A Systematic Review and Meta-Analysis. J. Oral Rehabil..

[24] miHabibi M., Nik T.H., Hooshmand T. (2007). Comparison of Debonding Characteristics of Metal and Ceramic Orthodontic Brackets to Enamel: An In-Vitro Study. Am. J. Orthod. Dentofac. Orthop..

[25] Harris A.M., Joseph V.P., Rossouw P.E. (1992). Shear Peel Bond Strengths of Esthetic Orthodontic Brackets. Am. J. Orthod. Dentofac. Orthop..

[26] Joseph V.P., Rossouw E. (1990). The Shear Bond Strengths of Stainless Steel and Ceramic Brackets Used with Chemically and Light-Activated Composite Resins. Am. J. Orthod. Dentofac. Orthop..

[27] Minervini G., Franco R., Marrapodi M.M., Di Blasio M., Isola G., Cicciù M. (2023). Conservative Treatment of Temporomandibular Joint Condylar Fractures: A Systematic Review Conducted According to PRISMA Guidelines and the Cochrane Handbook for Systematic Reviews of Interventions. J. Oral Rehabil..

[28] Inchingolo A.D., Malcangi G., Semjonova A., Inchingolo A.M., Patano A., Coloccia G., Ceci S., Marinelli G., Di Pede C., Ciocia A.M. (2022). Oralbiotica/Oralbiotics: The Impact of Oral Microbiota on Dental Health and Demineralization: A Systematic Review of the Literature. Children.

[29] Odegaard J., Segner D. (1988). Shear Bond Strength of Metal Brackets Compared with a New Ceramic Bracket. Am. J. Orthod. Dentofac. Orthop..

[30] Kitahara-Céia F.M.F., Mucha J.N., Marques dos Santos P.A. (2008). Assessment of Enamel Damage after Removal of Ceramic Brackets. Am. J. Orthod. Dentofac. Orthop..

[31] Wang W.N., Meng C.L., Tarng T.H. (1997). Bond Strength: A Comparison between Chemical Coated and Mechanical Interlock Bases of Ceramic and Metal Brackets. Am. J. Orthod. Dentofac. Orthop..

[32] Lucchese A., Porcù F., Dolci F. (2001). Effects of Various Stripping Techniques on Surface Enamel. J. Clin. Orthod..

[33] Bishara S.E., Olsen M.E., VonWald L., Jakobsen J.R. (1999). Comparison of the Debonding Characteristics of Two Innovative Ceramic Bracket Designs. Am. J. Orthod. Dentofac. Orthop..

[34] Minervini G., Marrapodi M.M., Cicciù M. (2023). Online Bruxism-related Information: Can People Understand What They Read? A Cross-Sectional Study. J. Oral Rehabil..

[35] Janiszewska-Olszowska J., Szatkiewicz T., Tomkowski R., Tandecka K., Grocholewicz K. (2014). Effect of Orthodontic Debonding and Adhesive Removal on the Enamel–Current Knowledge and Future Perspectives—A Systematic Review. Med. Sci. Monit. Int. Med. J. Exp. Clin. Res..

[36] Boyer D.B., Engelhardt G., Bishara S.E. (1995). Debonding Orthodontic Ceramic Brackets by Ultrasonic Instrumentation. Am. J. Orthod. Dentofac. Orthop..

[37] Brosh T., Kaufman A., Balabanovsky A., Vardimon A.D. (2005). In Vivo Debonding Strength and Enamel Damage in Two Orthodontic Debonding Methods. J. Biomech..

[38] Oliver R.G., Griffiths J. (1992). Different Techniques of Residual Composite Removal Following Debonding--Time Taken and Surface Enamel Appearance. Br. J. Orthod..

[39] Almudhi A., Aldeeri A., Aloraini A.A.A., Alomar A.I.M., Alqudairi M.S.M., Alzahrani O.A.A., Eldwakhly E., AlMugairin S. (2023). Comparison of Enamel Surface Integrity after De-Bracketing as Affected by Seven Different Orthodontic Residual Cement Removal Systems. Diagnostics.

[40] Isacco C.G., Ballini A., De Vito D., Nguyen K.C.D., Cantore S., Bottalico L., Quagliuolo L., Boccellino M., Di Domenico M., Santacroce L. (2021). Rebalancing the Oral Microbiota as an Efficient Tool in Endocrine, Metabolic and Immune Disorders. Endocr. Metab. Immune Disord.-Drug Targets.

[41] Malcangi G., Patano A., Morolla R., De Santis M., Piras F., Settanni V., Mancini A., Di Venere D., Inchingolo F., Inchingolo A.D. (2023). Analysis of Dental Enamel Remineralization: A Systematic Review of Technique Comparisons. Bioengineering.

[42] Malcangi G., Inchingolo A.D., Patano A., Coloccia G., Ceci S., Garibaldi M., Inchingolo A.M., Piras F., Cardarelli F., Settanni V. (2022). Impacted Central Incisors in the Upper Jaw in an Adolescent Patient: Orthodontic-Surgical Treatment—A Case Report. Appl. Sci..

[43] Eichenberger M., Iliadi A., Koletsi D., Eliades G., Verna C., Eliades T. (2019). Enamel Surface Roughness after Lingual Bracket Debonding: An In Vitro Study. Materials.

[44] Webb B.J., Koch J., Hagan J.L., Ballard R.W., Armbruster P.C. (2016). Enamel Surface Roughness of Preferred Debonding and Polishing Protocols. J. Orthod..

[45] Goldoni R., Dolci C., Boccalari E., Inchingolo F., Paghi A., Strambini L., Galimberti D., Tartaglia G.M. (2022). Salivary Biomarkers of Neurodegenerative and Demyelinating Diseases and Biosensors for Their Detection. Ageing Res. Rev..

[46] Heravi F., Shafaee H., Abdollahi M., Rashed R. (2015). How Is the Enamel Affected by Different Orthodontic Bonding Agents and Polishing Techniques?. J. Dent..

[47] The PRISMA Statement for Reporting Systematic Reviews and Meta-Analyses of Studies That Evaluate Healthcare Interventions: Explanation and Elaboration–PubMed. https://pubmed.ncbi.nlm.nih.gov/19622552/.

[48] Dumbryte I., Malinauskas M. (2021). In Vivo Examination of Enamel Microcracks after Orthodontic Debonding: Is There a Need for Detailed Analysis?. Am. J. Orthod. Dentofac. Orthop..

[49] Caixeta R.V., Berger S.B., Lopes M.B., Paloco E.A.C., Faria É.M., Contreras E.F.R., Gonini-Júnior A., Guiraldo R.D. (2021). Evaluation of Enamel Roughness after the Removal of Brackets Bonded with Different Materials: In Vivo Study. Braz. Dent. J..

[50] Faria É.M., Guiraldo R.D., Berger S.B., Correr A.B., Correr-Sobrinho L., Contreras E.F.R., Lopes M.B. (2015). In-Vivo Evaluation of the Surface Roughness and Morphology of Enamel after Bracket Removal and Polishing by Different Techniques. Am. J. Orthod. Dentofac. Orthop..

[51] Gorucu-Coskuner H., Atik E., Taner T. (2018). Tooth Color Change Due to Different Etching and Debonding Procedures. Angle Orthod..

[52] Pinzan-Vercelino C.R.M., Souza Costa A.C., Gurgel J.A., Salvatore Freitas K.M. (2021). Comparison of Enamel Surface Roughness and Color Alteration after Bracket Debonding and Polishing with 2 Systems: A Split-Mouth Clinical Trial. Am. J. Orthod. Dentofac. Orthop..

[53] Malekpour B., Ajami S., Salehi P., Hamedani S. (2022). Use of Nano-Hydroxyapatite Serum and Different Finishing/Polishing Techniques to Reduce Enamel Staining of Debonding after Orthodontic Treatment: A Randomized Clinical Trial. J. Orofac. Orthop./Fortschritte Der Kieferorthopädie.

[54] Karamouzos A., Zafeiriadis A.A., Kolokithas G., Papadopoulos M.A., Athanasiou A.E. (2019). In Vivo Evaluation of Tooth Colour Alterations during Orthodontic Retention: A Split-Mouth Cohort Study. Orthod. Craniofac. Res..

[55] Ye C., Zhao Z., Zhao Q., Du X., Ye J., Wei X. (2013). Comparison of Enamel Discoloration Associated with Bonding with Three Different Orthodontic Adhesives and Cleaning-up with Four Different Procedures. J. Dent..

[56] Janiszewska-Olszowska J., Tomkowski R., Tandecka K., Stepien P., Szatkiewicz T., Sporniak-Tutak K., Grocholewicz K. (2016). Effect of Orthodontic Debonding and Residual Adhesive Removal on 3D Enamel Microroughness. PeerJ.

[57] Nimplod P., Tansalarak R., Sornsuwan T. (2021). Effect of the Different Debonding Strength of Metal and Ceramic Brackets on the Degree of Enamel Microcrack Healing. Dent. Press J. Orthod..

[58] Sedky Y., Gutknecht N. (2018). The Effect of Using Er,Cr:YSGG Laser in Debonding Stainless Steel Orthodontic Brackets: An in Vitro Study. Lasers Dent. Sci..

[59] Yassaei S., Aghili H., Joshan N. (2015). Effects of Removing Adhesive from Tooth Surfaces by Er:YAG Laser and a Composite Bur on Enamel Surface Roughnessand Pulp Chamber Temperature. Dent. Res. J..

[60] Al Maaitah E.F., Abu Omar A.A., Al-Khateeb S.N. (2013). Effect of Fixed Orthodontic Appliances Bonded with Different Etching Techniques on Tooth Color: A Prospective Clinical Study. Am. J. Orthod. Dentofac. Orthop..

[61] Iglesias A., Flores T., Moyano J., Artés M., Botella N., Gil J., Puigdollers A. (2023). Enamel Evaluation after Debonding of Fixed Retention and Polishing Treatment with Three Different Methods. Materials.

[62] Rix D., Foley T.F., Mamandras A. (2001). Comparison of Bond Strength of Three Adhesives: Composite Resin, Hybrid GIC, and Glass-Filled GIC. Am. J. Orthod. Dentofac. Orthop..

[63] Merone G., Valletta R., De Santis R., Ambrosio L., Martina R. (2009). A novel bracket base design: Biomechanical stability. Eur. J. Orthod..

[64] Al Shamsi A.H., Cunningham J.L., Lamey P.J., Lynch E. (2007). Three-Dimensional Measurement of Residual Adhesive and Enamel Loss on Teeth after Debonding of Orthodontic Brackets: An in-Vitro Study. Am. J. Orthod. Dentofac. Orthop..

[65] Karan S., Kircelli B.H., Tasdelen B. (2010). Enamel Surface Roughness after Debonding. Angle Orthod..

[66] Bollen C.M., Lambrechts P., Quirynen M. (1997). Comparison of Surface Roughness of Oral Hard Materials to the Threshold Surface Roughness for Bacterial Plaque Retention: A Review of the Literature. Dent. Mater..

[67] Ijbara M., Wada K., Tabata M.J., Wada J., Inoue G., Miyashin M. (2018). Enamel Microcracks Induced by Simulated Occlusal Wear in Mature, Immature, and Deciduous Teeth. Biomed. Res. Int..

[68] Suliman S.N., Trojan T.M., Tantbirojn D., Versluis A. (2015). Enamel Loss Following Ceramic Bracket Debonding: A Quantitative Analysis in Vitro. Angle Orthod..

[69] Cesur E., Arslan C., Orhan A.I., Bilecenoğlu B., Orhan K. (2022). Effect of Different Resin Removal Methods on Enamel after Metal and Ceramic Bracket Debonding: An in Vitro Micro-Computed Tomography Study. J. Orofac. Orthop./Fortschritte Der Kieferorthopädie.

[70] Ghafari J. (1992). Problems Associated with Ceramic Brackets Suggest Limiting Use to Selected Teeth. Angle Orthod..

[71] Oralbiotica/Oralbiotics: The Impact of Oral Microbiota on Dental Health and Demineralization: A Systematic Review of the Literature–PubMed. https://pubmed.ncbi.nlm.nih.gov/35883998/.

[72] Anaraki S.N., Shahabi S., Chiniforush N., Nokhbatolfoghahaei H., Assadian H., Yousefi B. (2015). Evaluation of the Effects of Conventional versus Laser Bleaching Techniques on Enamel Microroughness. Lasers Med. Sci..

[73] Pont H.B., Özcan M., Bagis B., Ren Y. (2010). Loss of Surface Enamel after Bracket Debonding: An In-Vivo and Ex-Vivo Evaluation. Am. J. Orthod. Dentofac. Orthop..

[74] Grzech-Leśniak K., Matys J., Żmuda-Stawowiak D., Mroczka K., Dominiak M., Brugnera Junior A., Gruber R., Romanos G.E., Sculean A. (2018). Er:YAG Laser for Metal and Ceramic Bracket Debonding: An In Vitro Study on Intrapulpal Temperature, SEM, and EDS Analysis. Photomed. Laser Surg..

[75] Almeida H.C., Vedovello Filho M., Vedovello S.A.S., Young A.A.A., Ramirez-Yañez G.O. (2009). ER: YAG Laser for Composite Removal after Bracket Debonding: A Qualitative SEM Analysis. Int. J. Orthod..

[76] Dostalova T., Jelinkova H., Remes M., Šulc J., Němec M. (2016). The Use of the Er:YAG Laser for Bracket Debonding and Its Effect on Enamel Damage. Photomed. Laser Surg..

[77] Mesaroș A., Mesaroș M., Buduru S. (2022). Orthodontic Bracket Removal Using LASER-Technology-A Short Systematic Literature Review of the Past 30 Years. Materials.

[78] Marchi R., De-Marchi L., Terada R., Terada H. (2012). Comparison between Two Methods for Resin Removing after Bracket Debonding. Dent. Press. J. Orthod..

[79] Al Shamsi A., Cunningham J.L., Lamey P.J., Lynch E. (2006). Shear Bond Strength and Residual Adhesive after Orthodontic Bracket Debonding. Angle Orthod..

